# Novel Indications for Bruton’s Tyrosine Kinase Inhibitors, beyond Hematological Malignancies

**DOI:** 10.3390/jcm7040062

**Published:** 2018-03-21

**Authors:** Robert Campbell, Geoffrey Chong, Eliza A. Hawkes

**Affiliations:** 1 Department of Medical Oncology and Clinical Haematology, Olivia Newton-John Cancer Research Institute, Austin Health, Heidelberg, VIC 3084, Australia; robert.campbell@austin.org.au (R.C.); geoff.chong@onjcri.org.au (G.C.); 2 Bendigo Cancer Centre, Bendigo Health, Bendigo, VIC 3084, Australia; 3 Department of Oncology, Northern Hospital, Melbourne, VIC 3084, Australia; 4 Department of Medical Oncology, Ballarat Health Services, Ballarat, VIC 3084, Australia; 5 Department of Medical Oncology, Eastern Health, Melbourne, VIC 3084, Australia; 6 Eastern Health, Monash University Clinical School, Melbourne, VIC 3084, Australia

**Keywords:** ibrutinib, Bruton’s tyrosine kinase, solid tumors

## Abstract

Bruton’s tyrosine kinase (BTK) is a critical terminal enzyme in the B-cell antigen receptor (BCR) pathway. BTK activation has been implicated in the pathogenesis of certain B-cell malignancies. Targeting this pathway has emerged as a novel target in B-cell malignancies, of which ibrutinib is the first-in-class agent. A few other BTK inhibitors (BTKi) are also under development (e.g., acalabrutinib). While the predominant action of BTKi is the blockade of B-cell receptor pathway within malignant B-cells, increasing the knowledge of off-target effects as well as a potential role for B-cells in proliferation of solid malignancies is expanding the indication of BTKi into non-hematological malignancies. In addition to the expansion of the role of BTKi monotherapy, combination therapy strategies utilizing ibrutinib with established regimens and combination with modern immunotherapy compounds are being explored.

## 1. Introduction

Bruton’s Tyrosine Kinase (BTK) is a critical terminal kinase enzyme in the B-cell antigen receptor (BCR) signaling pathway. The BTK gene is located on the X chromosome (Xq21.33-Xq22) and was first described in the setting of X-linked agammaglobulinemia, a disorder characterized by a lack of maturation and development of B lymphocytes and gammaglobulins due to a mutation in BTK [1,2]. Treatment with lifelong immune globulin replacement is standard therapy [2], and apart from a susceptibility to infections, patients tend to have good quality of life into adulthood and lead productive lives [3], implying that inhibition of this pathway is likely to be tolerable in clinical practice. BTK activation has been implicated in the pathogenesis of certain B-cell malignancies [4]. Subsequently, targeting this pathway emerged as a novel target in B-cell malignancies [5]. Ibrutinib (formerly PCI-32765; Janssen, Janssen Pharmaceutica, Beerse, Belgium) was the first in class to come into mainstream clinical practice. Other BTK inhibitors under evaluation include acalabrutinib (ACP 196), tirabrutinib (ONO/GS 4059), zanubrutinib (BGB 3111), spebrutinib (AVL-292, CC-292) and CGI-1746.

**Figure A1:**
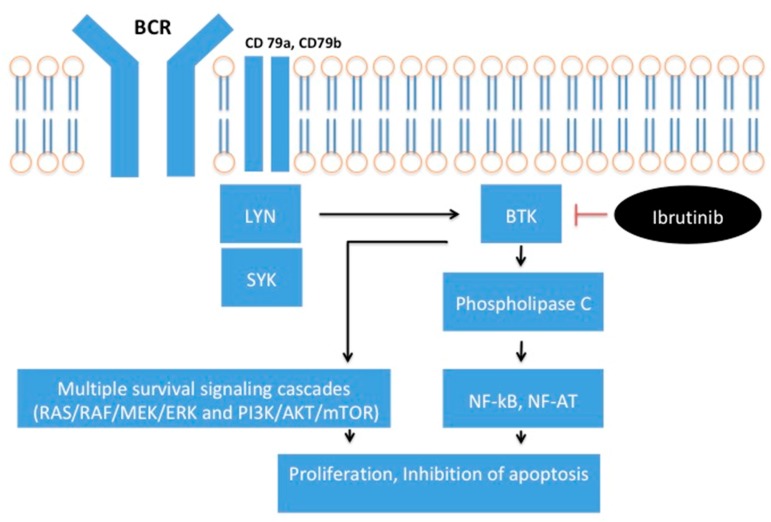
Simplified diagram of effects of BCR pathway activation. BCR activation leads to BTK plasma membrane translocation and phosphorylation by members of the Src kinases (e.g., LYN, SYK). Induction of BTK triggers activation of phospholipase C and calcium mobilization. This leads to downstream events such as proliferation and differentiation mediated through multiple transcriptions factors (NF-κB, NF-AT) and survival signaling cascades (RAS/RAF/MEK/ERK and PI3K/AKT/mTOR). Ibrutinib binds to BTK to inhibit BTK signaling.

**Figure A2:**
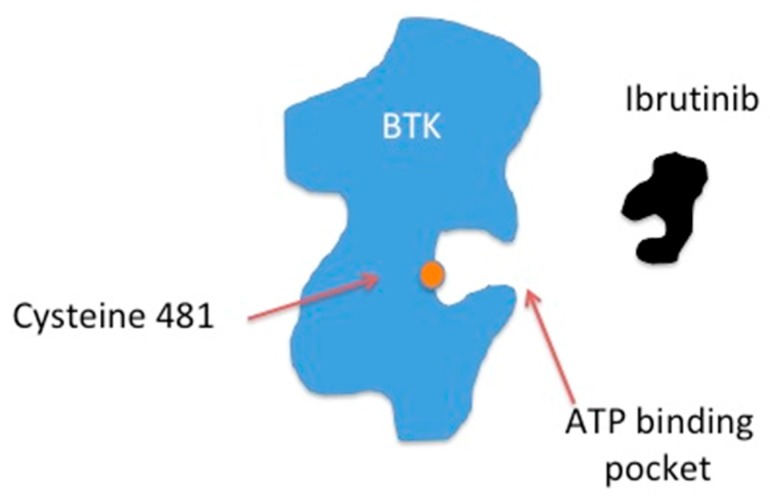
Ibrutinib is a covalent inhibitor of BTK that binds irreversibly to the cysteine 481 residue near the ATP binding pocket of BTK.

Bruton’s Tyrosine Kinase is a member of the Tec tyrosine kinase family and is involved in BCR signaling [6]. BCR activation leads to BTK plasma membrane translocation and phosphorylation by members of the Src kinases (e.g., LYN, SYK) [7]. Induction of BTK then triggers activation of phospholipase C and calcium mobilization. This leads to downstream events such as proliferation and differentiation mediated through multiple transcription factors (e.g., NF-κB, NF-AT) and survival signaling cascades (RAS/RAF/MEK/ERK and PI3K/AKT/mTOR) [1], Figure A1 (Appendix A). Ibrutinib is a covalent inhibitor of BTK that binds irreversibly to a cysteine residue (Cys-481) near the ATP binding pocket of BTK, Figure A2 (Appendix A) [8]. Ibrutinib has a half maximal inhibitory concentration (IC_50_) of BTK of 0.5 nM [8]. Sequence alignments show that 10 kinases in the human genome have a cysteine residue at an analogous position. These include BLK, BTK, BMX, EGFR, HER2, HER4, ITK, JAK3, TEC and TXK [9].

Chronic activation of the B-cell Receptor pathway is a key driver of B-cell non-Hodgkin lymphomas and hence, downstream blockade of this pathway utilizing BTK inhibitors (BTKi) in these diseases has proven very effective to date. Chronic lymphocytic leukemia (CLL) was the first malignancy where ibrutinib has become a critical component of standard therapy [10]. Ibrutinib has also been US Food and Drug Administration (FDA) and European Medicines Agency (EMA) approved for the treatment of relapsed mantle cell lymphoma. Ibrutinib efficacy is currently being explored in most B-cell malignancies including diffuse large B-cell lymphoma [11], follicular lymphoma [12], lymphoplasmacytic lymphoma [13], multiple myeloma [14], and primary CNS lymphoma [15]. It has also been studied in acute myeloid leukemia [16].

While the predominant mechanism of action of BTKi is blockade of the B-cell Receptor pathway within malignant B-cells, increasing knowledge of off-target effects as well as a potential role for B-cells in proliferation of solid malignancies is expanding the indication of BTKi beyond B-cell malignancies. Research has extended into the sphere of non-hematological malignancies where BTKi are currently being explored. These other potential targets are not altogether unexpected with BTKi given the established effect on multiple kinases central to cancer growth. There is precedent for such repurposing in other malignancies. The prototype example of this is imatinib, a drug originally developed for the breakpoint cluster region/Abelson murine leukemia viral oncogene homolog 1 (BCR-ABL)—driven chronic myeloid leukemia, being found to be active against mast/stem cell growth factor receptor (KIT) driven gastrointestinal stromal tumors (GIST) [17]. In addition to an expansion of the role of BTKi in non-B-cell malignancies, combination therapy strategies utilizing ibrutinib with established cytotoxic regimens as well as more modern immunotherapy compounds are also being explored. This topic provides an update since the earlier paper by Berglof et al. [18], and with a focus on the transition to clinical practice.

## 2. Ibrutinib in Specific Tumor Subtypes

Ibrutinib activity has been explored in several tumor types either as a single agent or in combination with other compounds. Clinical and pre-clinical data by tumor type will be discussed by tumor type and current clinical trials are summarized in Table A1 (Appendix A).

### 2.1. Lung Cancer

Epidermal growth factor receptor (*EGFR*) mutated non-small cell lung cancer (NSCLC) comprises approximately 15–20% of non-small cell lung cancer in white populations with higher rates in the order of 22–62% reported in Asian populations [19,20]. EGFR-directed tyrosine kinase inhibitors such as gefitinib and erlotinib have been the historical mainstay of initial therapy, although median overall survival has been in the order of 22–26 months [21,22]. Osimertinib, a third generation EGFR-directed TKI has shown promising first line data, as well as second line data in those who progress on first line treatment that harbor a *T790M* resistance mutation (more than 50% of cases) [23,24]. Ibrutinib has demonstrated pre-clinical efficacy in *EGFR*-mutated NSCLC. It has been shown to have high activity against *EGFR* mutated (*L858R*) cells, with moderate activity to *T790M* mutated cells [25,26]. Subsequent analysis showed no detectable expression of BTK and efficacy appeared to be due to an anti-EGFR effect [26]. Ibrutinib was shown to arrest cell lines in a G0/G1 phase as well as induce apoptosis [25]. A more recent study confirmed that ibrutinib inhibits mutant *EGFR* kinases through formation of a covalent bond with Cys797 similar to other irreversible EGFR TKIs such as gefitinib and erlotinib [27]. This indicates the efficacy of ibrutinib in NSCLC is an off-target effect, rather than through BTK inhibition. The ibrutinib activity against *EGFR* mutant cell lines was similar to that of erlotinib in one comparative study, but also with preserved activity against *T790M* mutated cell lines which were not sensitive to erlotinib [26]. This effect was not seen in non-mutated *EGFR* expressing lung cancer cells. Tumor progression was slowed by ibrutinib in xenograft mouse models, however not to same extent as seen with gefitinib [25]. Ibrutinib also slowed tumor progression in a *T790M* mutation mouse model. A combination of ibrutinib and trametinib (a *BRAF* inhibitor) showed promise in cell line research, however did not translate to a benefit in xenograft mouse models [25]. One research group proposed that a specially designed formulation or dosage of ibrutinib, or alteration of the pharmacokinetic property itself may achieve improved efficacy in clinical application as binding to EGFR is less efficient than other irreversible EGFR inhibitors, such as gefitinib or erlotinib [27]. Based on the preclinical efficacy signals, ibrutinib is undergoing evaluation in a phase I/II trial in previously-treated *EGFR*-mutant NSCLC (clinicaltrials.gov ID: NCT02321540). Its future role in this subtype of lung cancer is unclear given the encouraging data of osimertinib in this clinical cohort [28].

### 2.2. Breast Cancer

Breast cancer is the most common cause of cancer death in women globally [29]. It is a heterogeneous disease, largely characterized by the presence of hormone receptors (estrogen and progesterone), and human epidermal growth factor receptor 2 (HER2). Studies of HER2 positive breast cancer cell lines have demonstrated ibrutinib causes reduction in phosphorylation of EGFR, HER2 and human epidermal growth factor receptor 3 (HER3) [30], effects that are off-target to its original purpose in BTK inhibition. This subsequently suppressed downstream signaling of AKT and MAPK. Ibrutinib was combined with the dual PI3K/mTOR inhibitor BEZ235 which showed synergism in decreasing cell viability. The high sensitivity of ibrutinib on HER2-positive breast cancer cell viability might be explained by an addiction of these cells to the HER2/PI3K/AKT/mTOR signaling pathway [30]. Separately, ibrutinib, as well as other BTK inhibitors spebrutinib and CGI- 1746, were shown to reduce breast cancer cell survival and prevent drug resistant clones from arising [31]. Ibrutinib impacted HER2+ breast cancer cell viability at lower concentrations than the already approved HER2 TKI lapatinib [31]. Developing on this, ibrutinib has been shown to inhibit growth in a HER2+ xenograft model—with reduction of phosphorylation of HER2, BTK, AKT, ERK and histone H3 and increasing cleaved caspase-3 signals [31]. This promising data has raised the possibility of ibrutinib having clinical utility as a targeted therapy in HER2+ve breast cancer.

A novel isoform of BTK (BTK-C) has also been described as expressed in several human breast cancer cell lines and tumor tissues at relatively low levels, yet still providing an important function in protecting breast cancer cells from apoptosis [32]. One study found that 30% of breast cancer tissues stain positively for BTK and that among 23 HER2-positive human breast cancer tissues 43% were positive for BTK with a statistically significant association of expression of BTK with HER2 expression. Noting this association, it has been hypothesized that ibrutinib may have further therapeutic utility in HER2-positive breast cancer by providing the opportunity to target both kinases simultaneously as this combination should occur in a significant percentage of HER2-positive breast cancers [31].

### 2.3. Gastro-Oesphageal Cancer

Metastatic gastric cancer and esophageal cancer is associated with a poor prognosis despite the availability of several cytotoxic regimens. Approximately 15% of Western patients with adenocarcinoma overexpress HER2, with trastuzumab being an option to incorporate into first line treatment in this setting [33]. There is modest benefit from therapy for relapsed disease. Options include further cytotoxic treatment, with the incorporation of ramucirumab, a vascular endothelial growth factor receptor-2 (VEGFR2) antagonist showing modest benefit [34]. Pembrolizumab, a programmed cell death 1 (PD-1) immunotherapy agent is also FDA approved after two or more lines of therapy in those that have programmed-cell death ligand-1 (PD-L1) positive tumors [35]. Effective treatment in this setting is an unmet clinical need.

BTK over-expression has been described in gastric carcinoma tissues and gastric carcinoma cells [36]. In comparison normal gastric cells expressed little BTK. In subsequent experiments, knockdown of BTK expression selectively inhibited the growth of gastric cancer cells, but not the normal mucosal epithelium [36]. Ibrutinib was shown to induce apoptosis in gastric cancer cells and to be a chemo-sensitizer to docetaxel. Decreased tumor growth and increased cell apoptosis in tumors formed in mice inoculated with gastric carcinoma cells was also seen. This action was attributed to an anti-proliferative effect. Further evaluation of pharmacokinetic and pharmacodynamic properties of Ibrutinib in gastric cancer are underway and the possibility of combination strategies was raised [36].

To identify new biomarker directed therapeutic approaches in esophageal cancer Chong et al. integrated genomic profiles of 17 esophageal tumor-derived cell lines with drug sensitivity data from small molecule inhibitor profiling [37]. Sensitivity to inhibition of BTK in *MYC* amplified esophageal tumor lines and this genetic dependency could be demonstrated with ibrutinib. Ibrutinib elicited G_1_ cell cycle arrest and apoptosis in both *MYC* and *HER2* amplified tumors, suggesting this drug could be used to treat biomarker-selected groups of patients with esophageal cancer. *MYC* amplification is described in 32% of esophageal adenocarcinomas and 23% of squamous cell carcinomas [38]. This research group have developed a phase II trial of ibrutinib in patients with MYC and/or HER2 amplified esophageal cancer is currently recruiting based on this (NCT02884453). In summary, some of the effects of ibrutinib in gastric and esophageal cancer may be attributable to BTK inhibition, whereas activity in HER2 amplified disease may be due to off-target activity. Further studies are required to clarify this.

### 2.4. Pancreatic Cancer

Pancreatic cancer is a highly aggressive malignancy, with pancreatic ductal adenocarcinoma (PDAC) accounting for 85% of cases. It is associated with a high mortality rate and is the fourth leading cause of cancer deaths in Europe [39]. BTK signaling appears to play roles in multiple pathways in pancreatic adenocarcinoma. Pancreatic ductal adenocarcinoma (PDAC) has a characteristic stromal fibro-inflammatory reaction that is an obstacle to effective therapy, rendering most pancreatic malignancies refractory to conventional chemotherapy. This stroma comprises multiple types of inflammatory cells. Mast cell infiltration has been correlated with higher tumor grade and inferior survival in PDAC [40]. BTK has been validated as playing a critical role for mast cell degranulation in mouse models of pancreatic cancer (including insulinoma and PDAC) [41]. Ibrutinib was found to lead to vasculature collapse and tumor regression in insulinoma and had an unexpected anti-fibrotic effect in PDAC—with synergism with standard chemotherapy extending survival in mouse models. BTK has also been described to regulate B-cell and macrophage mediated T-cell suppression in pancreatic adenocarcinoma [42]. Ibrutinib was shown to restore T cell-dependent anti-tumor immune responses to inhibit PDAC growth and improve chemotherapy responsiveness. These studies suggest an on-target BTK mediated effect of ibrutinib in pancreatic cancer. A phase II/III trial is currently evaluating ibrutinib versus placebo in combination with standard nab-paclitaxel/gemcitabine chemotherapy in patients with previously untreated metastatic pancreatic cancer [43] (NCT 02436668).

### 2.5. Ovarian Cancer

Ovarian cancer is the second most common gynecological malignancy in developed countries [44]. Platinum-based chemotherapy is the cornerstone of systemic treatment for this condition and platinum sensitivity and the platinum free interval have remained critical to deciding subsequent systemic treatments in the relapsed setting [45]. In ovarian cancer patients, increased BTK expression correlated with the presence of advanced stage disease and an increased chance of metastasis [46]. In the same study, those with moderate or intense staining had significantly inferior survival rate than those with weak or focal staining. It has been proposed that ovarian cancer stem cells play a role in cisplatin resistance and that BTK mediates chemoresistance through regulation of ovarian cancer stem cells. Loss of function studies demonstrated that BTK knockdown led to reduced expression of JAK2 and STAT3 targets—with comparable efficacy to BTK knockdown demonstrated with ibrutinib treatment. This in turn decreased cancer cell survival through *Sox*-2 and *BCL*-*XL* genes. Cell viability testing was performed after treatment with cisplatin alone, ibrutinib alone or a combination of both. Synergistic effects of the combination of ibrutinib plus cisplatin were demonstrated on serous and clear cell types of ovarian cancers. The synergistic effect suggested ibrutinib potentially sensitizes cancer cells to platinum, and it was postulated that each drug also had independent activity. The authors of the study concluded that ibrutinib may be a candidate drug to attempt to overcome platinum resistance in clear cell and malignant cystadenocarcinoma, via an on-target BTK effect.

### 2.6. Prostate Cancer

As with other adenocarcinomas, BTK expression has also been described in prostate cancer cells, with the BTK-C isoform predominantly expressed [47]. In prostate cancer cell lines, down regulation of this protein with RNA interference or inhibition with BTKi, ibrutinib, spebrutinib and CGI-1746, all decreased cell survival and induced apoptosis via an on-target BTK mediated effect [47]. Microarray results show that inhibiting BTK increases expression of the apoptosis-related marker Asp175. Overexpression of BTK-C is associated with elevated expression of genes with functions related to cell adhesion, cytoskeletal structure, and extracellular matrix. This is consistent with studies that show BTK signaling is important for adhesion and migration of B-cells and suggest that BTK-C may confer similar properties in prostate cancer cells to those in mature B-cells. BTK-C may represent both a potential biomarker and novel therapeutic target for prostate cancer. A phase I/II study is evaluating neoadjuvant ibrutinib in men with localized prostate cancer undergoing radical prostatectomy as their initial curative intent treatment (NCT02643667).

### 2.7. Glioma

Glioblastoma multiforme (GBM) is the most common primary brain tumor, with currently no effective targeted therapies. Chemoradiotherapy is central to the management of this disease, however, prognosis remains poor, with limited cytotoxic choices due to the necessity of blood-brain barrier penetration of any therapeutic. The prognostic significance of BTK expression in glioma has been studied, with high expression of BTK proposed as a novel prognostic marker [48]. Bioinformatics data indicate that BTK is significantly higher in clinical glioma samples compared to normal brain cells, with higher levels of BTK mRNA described in GBM compared with normal brain and astrocyte samples [49]. Ibrutinib was shown to inhibit proliferation, migration, and invasion ability of glioma cells in cell lines and a xenograft mouse model [48]. As demonstrated in other solid tumor types, ibrutinib induced G1 cell-cycle arrest by regulating multiple cell cycle-associated proteins. BTK also appeared to be required for EGFR-induced NF-κB activation in glioma cells. Another study has described an antitumor effect that induces autophagy through AKT/mTOR signaling pathway in GBM cells [50]. In xenograft mouse models, tumorigenesis was significantly reduced in BTK silenced or ibrutinib-treated mice compared to controls [49]. Separately, it was demonstrated that temozolomide (TMZ) treatment is associated with an AKT-induced NF-κB activation, which leads to decreased sensitivity and protection against TMZ [51]. Combining TMZ with ibrutinib is a logical combination warranting further study. Importantly, in patients with central nervous system lymphomas, ibrutinib has been proven to effectively cross the blood brain barrier, a prerequisite for treatment of any brain cancer [52]. Most of the mechanisms of ibrutinib efficacy described are on-target effects of BTK inhibition. The EGFR-induced NF-κB activation may be attributable to BTK pathways—however this is not currently clear given the molecular mechanism of EGFR induced activation of NF-κB in glioma is currently unclear [48].

### 2.8. Renal Cell Carcinomas

Current treatment options for metastatic renal cell carcinoma (RCC) include molecularly targeted agents or immunotherapy. Molecularly targeted agents include drugs that predominantly focus on vascular endothelial growth factor (VEGF) pathways—such as sorafenib, sunitinib, pazopanib, axitinib and cabozantinib or agents that target the mechanistic target of rapamycin (mTOR) pathway such as everolimus and temsirolimus [53]. Established immunotherapy strategies include checkpoint inhibition with single agent PD-1 therapy [54], and more recent data include combined checkpoint inhibition incorporating cytotoxic T-lymphocyte-associated antigen 4 (CTLA-4) therapy in addition to PD-1 therapy (NCT02231749), with high dose interleukin-2 now a historical standard. BTK has not been well described in RCC, however interleukin-2-inducible T cell kinase (ITK) is also inhibited by ibrutinib (an off-target effect) and has been described to have immune-modulatory effects (see novel BTK inhibitor combinations below). Hosier et al., describe a case of a 22-month ongoing response to ibrutinib in a patient with RCC who was treated with ibrutinib for concurrent CLL [55]. Ibrutinib is currently being studied in a phase Ib/II trial in conjunction with nivolumab in previously treated metastatic kidney cancer (NCT02899078).

## 3. Novel BTK Inhibitor Combinations

Ibrutinib has entered clinical care for multiple lymphoid malignancies, and the toxicity is well established. Other next-in-class BTKi have similar adverse event profiles. Unlike the majority of multi-target tyrosine kinase inhibitors, ibrutinib causes minimal rash, only modest gastrointestinal and rheumatological toxicities, with infection, myelosuppression, cardiac arrhythmias, and anti-platelet activity the predominant toxicities. These side effects are proving to be manageable.

In lymphoid malignancies, BTKi have been safely combined with multiple different chemotherapeutic regimens and monoclonal antibodies such as rituximab and ofatumumab [56,57,58].

The known off-target effects of BTKi, in addition to the role of inhibiting host immune B-cells in the tumor microenvironment married with the attractive side effect profile, and knowledge of safe use with multiple other therapies provide good rationale for novel combinations in solid tumors.

As mentioned above, ibrutinib synergizes with chemotherapy in pre-clinical models of pancreatic, ovarian, and neurological cancers. It also potentially synergizes with other agents such as checkpoint inhibitors—such as PD-1 inhibitors. In healthy cells, PD-1 blockade inhibits B-cell receptor mediated signaling, and more understanding of this interaction in malignant cells is required [59].

The tumor microenvironment is a complex process where many tumors actively suppress various immune mechanisms to protect the tumor. Tec family kinases play a role in regulating the tumor microenvironment.

Ibrutinib inhibits (ITK), an enzyme important for the survival of Th2 T cells and therefore might have immunomodulatory effects in addition to its BTK activity by shifting the balance between Th1 and Th2 T cells [60]. The shift towards Th2 differentiation leads to less maturation of cytotoxic T lymphocytes, and the release of immunosuppressive cytokines such as IL-10 and transforming growth factor beta (TGF-β) in the tumor microenvironment [61]. This environment also can downregulate dendritic cells and hence adequacy of a cellular immune response [62].

Emerging data from various mouse models of cancer suggest that ibrutinib may have immunological effects by affecting myeloid-derived suppressor cells. Myeloid-derived suppressor cells (MDSCs) are a heterogeneous group of immature myeloid cells and expansion has been linked to loss of immune effector cell function and reduced efficacy of immune-based cancer therapies [63]. MDSCs commonly express BTK and are present in the tumor microenvironment of many different cancers. MDSCs are associated with immunosuppression leading to evasion of anti-tumor immune responses [64]. Treatment of mice bearing mammary tumors with ibrutinib resulted in reduced frequency of MDSCs in both the spleen and tumor [63]. It also resulted in a significant reduction of MDSCs in wild-type mice bearing melanoma tumors suggesting that BTK inhibition plays an important role in an observed reduction of MDSCs in vivo. In mice inoculated with mammary cancer cells, mice treated with combination of ibrutinib and anti-PDL1 produced a significant reduction in tumor growth compared with either agent alone [63].

Combination of Ibrutinib and anti-PD-L1 antibody suppressed tumor growth in mouse models of lymphoma that are intrinsically insensitive to ibrutinib [65]. The combined effect of the two agents was also documented for some models of solid tumors including triple negative breast cancer and colon cancer [65]. This was accompanied by enhanced antitumor T-cell responses. PD1/PDL-1 blockade and ibrutinib is now being explored in hematological malignancies [NCT02795182]. In solid tumors this combination is also the subject of several active clinical trials of colon cancer, melanoma, renal cell carcinoma, non-small cell lung cancer, breast cancer and pancreatic cancer (NCT03332498, NCT02899078, NCT03021460, NCT02403271). This combination should potentially be evaluated more broadly, even if tumors do not express BTK.

## 4. Discussion

BTK inhibitors are an established therapy in the treatment of numerous lymphoid malignancies. However, pre-clinical studies with BTKi in non-B-cell cancers show efficacy due to numerous mechanisms. Some effects are attributed to BTK signaling pathways, such as the inflammatory stroma of pancreatic cancer previously described and have furthered our understanding and knowledge of these processes. In several tumor subtypes such as lung, breast and esophageal, BTKi efficacy appears to be via other intracellular signals with a corresponding cysteine residue in the ATP binding site demonstrating that ibrutinib is not entirely specific in its binding to BTK. Relative binding affinity of ibrutinib to signals within 10 other kinase pathways (BTK, ITK, TEC, BMX, TXK, BLK, JAK3, EGFR, HER2, HER4) has been described [18]. The possible effects of BTKi on the immune system via their effect on non-malignant mature B-cells, myeloid-derived suppressor cells and its exploration in combination with targets of immune checkpoint inhibition (PD1/PDL-1) add a further layer of complexity to the role of BTKi.

The growing evidence of BTKi activity on signaling pathways beyond the original intended clinical application of BTK signal blockade highlights the importance of ongoing thorough laboratory research in testing of compounds to evaluate their full clinical potential. Pursuit of such research beyond drug registration for one class of diseases is necessary to ensure potential targets are identified and exploited. Such drug development has been shown to lead to clinically meaningful repurposing of established therapies. Haura and Rix describe potential methods to achieve this [66]. They proposed compound screening against panels of cancer cell lines with well-characterized genetics and underlying biology that may uncover potential off-target effects [67,68]. Another approach would be systematically defining and characterizing off-target compounds with modern chemical biology techniques including chemical proteomics, yeast three hybrid and microarray-based analyses [66]. These processes are often not undertaken due to the significant financial and experimental effort involved.

The data demonstrating BTKi activity in non-hematological malignancies has been predominantly pre-clinical to date with only a small number of clinical studies published. Given the mechanisms of action vary in the varying tumor subtypes, future evaluation of BTKi in solid malignancies will require tumor specific trials, as the rationale of combinations will differ. Table A1 (Appendix A) lists current clinical trials utilizing ibrutinib either as single agent therapy or in combination with other regimens in non-hematological malignancies. Tailored in vivo biomarker research incorporated into clinical trials harnessing these novel mechanisms of action is imperative to advance the knowledge of these off-target effects [43]. Caution should be employed when developing combinations of BTKi with novel agents for clinical evaluation due to the potential for negative efficacy interactions or synergistic toxic effects from off-target activity.

Ibrutinib is the most established of the BTKi, and next-in-class novel BTK inhibitors continue to develop. These include acalabrutinib (ACP 196), tirabrutinib (ONO/GS 4059), zanubrutinib (BGB 3111), spebrutinib (AVL-292, CC-292) and CGI-1746. These compounds have different affinities and hence likely different actions than those demonstrated with ibrutinib. Acalabrutinib, a second generation BTK inhibitor, has been granted accelerated FDA approval based on preliminary results of a phase II trial in mantle cell lymphoma (NCT02213926). Little is known about the diversity of action of these novel BTKi on other intracellular signaling pathways. Based on the knowledge of ibrutinib, these newer agents will also require ongoing studies.

## 5. Conclusions

Ibrutinib has demonstrated encouraging preclinical activity in a wide range of non-hematological malignancies. Some of these effects relate directly to BTK signaling, furthering our understanding of this complicated pathway and its multitude of effects in cell physiology and pathology. Yet many studies have proven BTKi activity in a multitude of other kinase pathways reliant on the cysteine residue binding and necessary for cancer cell survival. This knowledge exposes potential avenues for expanding the clinical utility of BTKi and provides a lesson for development of other novel agents that may have valuable hidden efficacy signals. Research driven by scientific, rather than commercial interests is desperately required to allow the full potential of new therapeutics to be achieved.

## Appendix A

**Table A1:** Table of current trials of ibrutinib in non-hematological malignancies.

Tumor (Subtype)	Phase	Setting	Combination Drug to Ibrutinib/Single Agent	Trial Reference
Non-Small Cell Lung Cancer (*EGFR* or HER2 mutation positive)	I/II	At least one line of EGFR directed therapy	Single agent	NCT02321540
Breast Cancer (HER 2)	I/II	Previous trastuzumab-emtansine	Trastuzumab	NCT03379428
Gastro-esophageal cancer (C-Myc and HER2 amplified)	II	At least one prior therapy	Single agent	NCT02884453
Pancreatic Cancer	I/II	First line of treatment for metastatic disease	Gemcitabine and nab-paclitaxel	NCT02562898
	II/III	First line of treatment for metastatic disease	Gemcitabine and nab-paclitaxel	NCT02436668
Prostate Cancer	I/II	Neoadjuvant prior to radical prostatectomy	Single agent	NCT02643667
Renal Cell Carcinoma	I/II	At least one prior therapy	Nivolumab	NCT02899078
Colorectal Cancer	I/II	Prior 5FU, irinotecan, oxaliplatin, and EGFR treatment if applicable	Pembrolizumab	NCT03332498
Melanoma	II	First line metastatic, or unresectable stage III	Pembrolizumab	NCT03021460
	II	Previous PD-1/PD-L1 therapy	Single agent	NCT02581930
Neuroendocrine tumor	II	Any line of treatment	Single agent	NCT02575300
Multiple tumor stream trials (Breast, Non-small cell lung cancer, Pancreatic cancer)	I/II	At least 2 lines of prior treatment for breast cancer, at least 1 line of treatment for NSCLC and pancreatic cancer	Durvalumab	NCT02403271
(Renal Cell, Urothelial, Gastric, Colorectal)	I/II	At least 2 lines of treatment for colorectal cancer; At least 1 line of systemic treatment for other tumor types	Everolimus (RCC cohort), Paclitaxel (Urothelial cohort), Docetaxel (gastric cohort), Cetuximab (colorectal cohort)	NCT02599324

Accessed via clinicaltrials.gov on 30 December 2017.
